# ‘Out of the frying pan into the fire’: a qualitative study of the impact on masculinity for men living with advanced prostate cancer

**DOI:** 10.1177/26323524231176829

**Published:** 2023-05-29

**Authors:** Yakubu Salifu, Kathryn Almack, Glenys Caswell

**Affiliations:** Lecturer in Palliative Care, International Observatory on End of Life Care, Division of Health Research, Faculty of Health and Medicine, University of Lancaster, Lancaster, LA1 4AT, UK; Professor of Family Lives and Care, Centre for Future Societies Research Communities, Young People and Family Lives Centre for Research in Public Health and Community Care, School of Health and Social Work, University of Hertfordshire, Hatfield, UK; Independent Social Researcher and Death Studies Scholar, Nottigham, UK

**Keywords:** advanced prostate cancer, African/Black men, culture, intersectionality, masculinity, men’s health, physical appearance, sexual life, social construction

## Abstract

**Background:**

Studies have highlighted how advanced prostate cancer causes biographical disruption and presents challenges to masculine identities for men. This article draws on a wider study that focused on the experiences of men living with advanced prostate cancer and their caregivers. Although men’s experience of advanced illness is not overlooked in the literature, only a small body of work has taken an in-depth look at men’s experiences with advanced prostate cancer and their caregivers in a non-Westernised cultural and social context.

**Objective:**

To explore how advanced prostate cancer impacts on men’s masculine identity from the perspective of patients and their caregivers.

**Methods:**

A qualitative study of men living with advanced prostate cancer (*n* = 23) and family caregivers (*n* = 23) in Ghana. We used the Consolidated Criteria for Reporting Qualitative Studies (COREQ) as the reporting guideline.

**Results:**

The findings from this study highlight profound challenges for most men to their masculine identities, from both the treatment and the symptoms of advanced prostate cancer within a non-Westernised, patriarchal society. Four main themes were developed. These were the impact on masculinity in terms of: (1) physical changes, (2) sexual ability, (3) socio-economic roles and (4) expressing emotions. Changes in physical appearance, feeling belittled, having no active sexual life and the inability to continue acting as provider and protector of the family made some men describe their situation as one of moving out of the ‘frying pan into the fire’.

**Conclusion:**

This study revealed the impact of advanced prostate cancer on masculine identity. These narratives add a new dimension to what is already known about the impacts on men’s masculine identities when dealing with advanced prostate cancer. This knowledge can help improve the care provided to men with advanced prostate cancer with emphasis on the cultures, beliefs and aspirations of these men and their caregivers.

## Introduction

Globally, prostate cancer is the most common male cancer and a leading cause of death in African men around the world.^1^ Prostate cancer incidence is higher among Black men than in other ethnicities.^2,3^ Chronic disease such as advanced prostate cancer causes fundamental changes in men’s everyday lives and leads to identity changes due to the disease’s unique trajectory and treatment side effects.^4–7^ Advanced prostate cancer therefore contributes to biographical disruption and presents challenges to masculine identities for men.^8–10^

Reviews of research and empirical studies exploring men’s response to prostate cancer in Western contexts have identified its impact on their masculinity.^11–16^ A significant subsection of this literature, however, focuses on men’s experiences of early-stage prostate cancer or life after treatment for prostate cancer.^17^ Some studies include the perspectives of African and Black men living in Western countries.^18,19^ We know very little about men’s experiences when facing advanced prostate cancer in non-Western contexts, particularly regarding challenges to their masculine identity. These unexplored aspects of the impact of prostate cancer highlight a critically important research gap for this population.

For Arrington,^20^ prostate cancer sheds light on, more than any other disease, the impact that illness can have for masculinity identities. Experiences of prostate cancer highlight intersections of chronic illness, culture, ageing and masculinity. Gender and masculinity are socio-cultural factors that influence health-related behaviours.^21^ Two review papers explore findings of studies of men’s experiences of prostate cancer that have included men of African and Caribbean origin, although all studies come from the United States or the United Kingdom.^17,22^ These reviews highlight a complex intersection of ethnicity with other factors in men’s experiences, including conceptualisations of masculinity. Many of the studies reviewed, however, do not have a specific focus on the impacts of (a) advanced prostate cancer and the associated impact on men’s masculine identities and (b) the applicability of these findings within the context of non-Westernised masculinity remains unclear. For example, in health resource-poor settings, there are further implications of managing the day-to-day living with, and treatment for, advanced prostate cancer due to other factors such as stigma^23^ and beliefs about what constitutes masculinity.

Connell^24^ suggests there is not one masculinity but many different masculinities. She defines masculinity as a social construction that is driven by culture, locality and particular historical periods.^24^ Conventionally, research into ‘gender and health’ has been women-centred.^13^ For instance, there has been extensive research on the impact of mastectomy on femininity, but there has been little research into the effect of prostate cancer surgical intervention (e.g. orchidectomy, the removal of the testes) on male gender identity.^25^ More recently, however, Robertson *et al.*^26^ identify the increasing attention being directed at understanding the concept of masculinities in relation to men’s experiences of health and ill-health. Robertson *et al.*^26^ assert that ‘masculinities can be recognised as both the producer and product of both structure and agency’ (p. 64). They suggest that masculinity could be experienced differently in diverse settings or even within the same person across time, hence the term *masculinities*. They argue that an understanding of masculinities is essential in research to assist clinicians and social scientists in developing ways to address the unique needs of men’s health. In addition, understanding masculinity(-ies) and its impact on health and illness will form part of public health agendas to create awareness and educate society on men’s health issues.

Over the last 20 years, gender has become a significant research focus in studies about African men in Africa on topics such as fatherhood and intimate partner violence. This provides a rich body of literature discussing issues of masculinity as a socially constructed, relational and hegemonic concept.^27^ Yet our understandings of how men’s lived experiences of masculinity affect and are affected by serious illness and healthcare practices remain primarily unexplored in Africa.^28,29^

Literature on caregivers’ accounts of caring for men living with prostate cancer is also limited. Where accounts exist, they are mostly from partners of patients and not the wider family caregivers such as adult children, siblings and parents. Furthermore, most studies are Western-based even if some include the experiences of Black men. The predominant focus is also on men in the early stages of prostate cancer, post-treatment or on prostate cancer survivors. A deeper understanding of how cultural codes of masculinity impact men’s experiences of disease and treatment in an African culturally specific context contributes a valuable perspective on masculinity and prostate cancer in a non-Westernised socio-cultural context with the views of diverse family caregivers including spouses. This study explored how prostate cancer impacts on men’s masculine identity from the perspective of patients and their caregivers.

## Methods

This qualitative study draws on data from a larger study that explored how men and their caregivers experienced living with advanced prostate cancer in a non-Western, patriarchal context.^30^ Sampling was purposive, and 23 men and their caregivers were recruited. Two rounds of interviews took place in the participants’ preferred place, which was usually at home.^31^ Twenty-three men and 23 caregivers participated in the first round of interviews. Four men died after the first interview, so follow-up interviews were carried out with the 19 surviving patients and their caregivers. In all, 63 interviews (21 joint/dyad interviews and 42 individual interviews) were conducted.^32^ The interviews were conducted in English (*n* = 11) or local dialect (*n* = 52) allowing the retention of quotes that are not easily translatable. Conducting a repeat interview ensured exploration of complex and sensitive issues surrounding masculinity while ensuring adequate and quality data.^33,34^ Data adequacy was achieved when data collected from 46 participants was sufficient in depth and breadth to understand the intersectionality of masculinity, physical appearance, sexuality, beliefs and values, emotionality through the experiences of men living with advanced prostate cancer and their family caregivers in Ghana. With participants’ permission, interviews were recorded to facilitate the capturing of accurate accounts of participants’ experiences on masculinity and living with or caring for advanced prostate cancer. An interview guide was developed based on the literature review, and research questions and researchers’ experiences guided the interview process. We report the characteristics of the men and their caregivers in Table 1.

**Table 1.** Characteristics of the 23 men and their family caregivers interviewed.

Men		Family caregivers	
Age (years)	Number	Relationship to patient	Number
49–58	6	Wife	10
59–68	10	Child	9
69–78	4	Mother	2
79–88	3	Sibling	2
Type of palliative treatment^a^		Years of care	
Hormone treatment	15	Less than 1	12
Surgical castration (2)/radical prostatectomy (4)	6	1–2	7
Radiation	4	3–4	3
No longer being actively treated	4	5+	1

a Some of the men received more than one treatment.

Data collection and analysis were done concurrently,^35^ and follow-up interviews allowed the interrogation of pertinent issues identified,^36^ especially around how advanced prostate cancer affects social norms of masculinity, as understood by participants. A qualitative data management software, NVivo, was used to manage the data. We read all transcripts and identified patterns in the data while interpreting and making sense of the impact of masculinity using thematic analysis.^37,38^ Four distinct but related themes about the impact of advanced prostate cancer on men’s sense of masculine identity were constructed based on participants’ perspectives. The Consolidated Criteria for Reporting Qualitative Studies (COREQ) was used as the reporting guideline.^39^

## Findings

**Figure 1.**
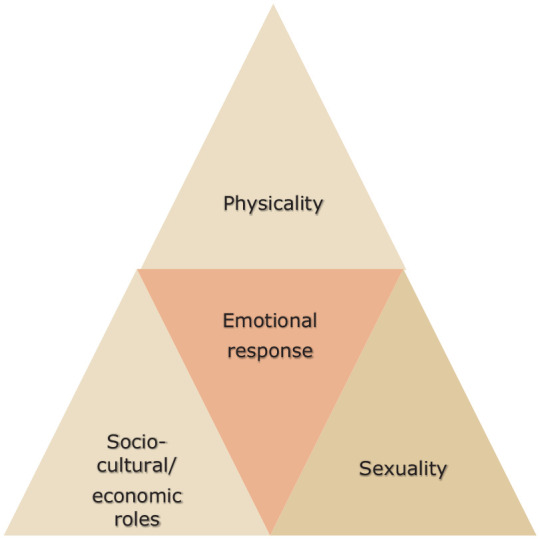
Cultural codes of masculinity in advanced prostate cancer.

The findings illustrate profound challenges, with men struggling with the impact of advanced prostate cancer on their masculinity in four main areas. The four themes are the impact of masculinity on men’s physicality, sexuality, socio-cultural roles and emotionality, as presented in Figure 1. The explanation of the themes is given in Table 2.

**Table 2.** The four key constructs of the impact of prostate cancer on masculinity.

Theme	Attributes/meaning
Physicality	This refers to the physical characteristics resulting from disruptive bodily changes such as chemotherapy-induced hair loss, enlarged breasts and fatigue/weakness that impact physical attributes associated with masculinity.
Sexuality	In this context, sexuality refers to the potency and the desire of the man to have penetrative sex, an idealised social construct of who a ‘real’ man should have as a sexual partner, as perceived by participants.
Socio-economic/cultural identity	This theme refers to the hegemonic ideals of men and the status they must occupy regardless of health or ill-health.
Emotionality	Hegemonic representations of masculinity include repression of certain emotions associated with being weak, such as crying and expressing fears. This theme refers to emotional responses related to changes in appearance, socio-cultural/economic status and declining sexual life.

### Masculinity and physicality

Just over half of the men expressed frustration related to their changing physical appearance, usually caused by side effects of chemotherapy and hormone replacement drugs. These changes included breast enlargement, loss of hair, weight loss and fragility:

Indeed, I use to be a well-built handsome man. I’ve wasted and changed so much . . . look [showing his hair] it’s sparse, and I look like a child with kwashiorkor [a malnourished child]. (Opoku, patient)

Here, Opoku implicitly refers to a diminished sense of a masculine identity as he feels he now looks more child-like in contrast to prior masculine ideal. Some perceived these changes to be feminising:

Hmm, I’m becoming a woman. After starting the chemo for some time, my breast had begun increasing just like that of ladies. If this should continue, it means I might need to wear a brassiere [he laughs]. (Nii, patient)

. . . now as you may have noticed my voice doesn’t sound like a man but sounds like that of a lady [high pitched] . . . That’s not man enough (Opoku, patient)

Both Nii and Opoku were concerned about physical changes resulting from chemotherapy that made them look like women. The use of terms such as *becoming a woman* or *not man enough* represents an embodied loss of masculinity.

Changing physical appearance was a concern for the men who were fastidious about their appearance and recognised that people may judge them by the way they look. Excessive weight loss resulting from advanced prostate cancer as well as a loss of strength and muscle wastage fractured their masculine appearance, and given this perception, their sense of their masculinity was damaged. Caregivers also noted that taking pride in their appearance was integral to men’s masculinity:

My husband is very particular about his physical appearance and the clothes he wears. As a man, one needs to look strong and healthy. His cancer treatment plus the disease has resulted in excessive weight loss. He is worried about the weight loss and his social status, but I reassured him. (Seiwaa, Daama’s wife)

Another important aspect related to their physicality is what men described as being *physically complete* and being seen as *real men*. This notion led to some men ruling out the option of certain treatments that doctors thought might be helpful for them, such as orchiectomy, which is the surgical removal of the testes. This procedure would lower androgen levels and reduce symptoms of prostate cancer. Some participants held the belief that testes symbolise manhood and contribute to the sexual prowess associated with masculine ideals. Therefore, some participants preferred not to have the surgery.

As for that operation [removal of the testes], I’ve ruled it out. It was out of the options for me from day one. Eii [exclamation] taking my testes out will mean I’m no longer a man. The balls make us men, you know. (Maegyida, patient)

Participants’ accounts illustrate that the appearance of the physical body is important in men’s perceptions of what it means to be masculine, and how the loss of male body parts may affect men’s sense of masculinity. The men struggled with their embodied loss of masculinity using the acceptable socially constructed and idealised body as a measure.

### Masculinity and sexuality

Issues related to the performance of (hetero) sexuality were common challenges that men recounted. The partial or complete loss of erectile function because of advanced prostate cancer had an impact on men’s sense of themselves as men. The challenges posed here included reduced sexual drive and impotence:

I’m overly concerned about my inability to have sex. Then what makes me a real man? You know a man is incomplete without it (his ability to have sex). You become the mockery of the town should your wife leak such information out. (Opoku, patient)

Hmmm, ‘meyɛ mmerɛ wo mpa mu’ (he whispered I’m impotent). When I reported to the hospital, they said this is usual with prostate cancer. I’ve been thinking about this, and for some time, I thought I was going mad because my manhood is gone. So, in effect, I am no longer a real man. (Kwakwa, patient)

Losing the ability to have sex caused some men to feel incomplete and subsequently affected their ‘manhood’ and, therefore, their dignity; for such men, having a libido, whatever their health status, is valued as a masculine trait. Otherwise, the man may be regarded as being impotent (*not man enough*) and incapable of satisfying a woman sexually:

My thing [penis] is not functioning. It’s not working [emphasis]. I mean, it is dead. Here I am. My dignity as a man is no more. I can’t pride myself as a complete man. (Maegyida, patient)

In some instances, the presence of an indwelling catheter could serve as a barrier preventing penetrative sex, even if the man could otherwise have sex. Some men believed because of the catheter, they could not fulfil their conjugal duty. This was in contradiction to cultural beliefs about men’s conjugal role in society:

I’ve never in my life experienced a ‘bad’ disease like prostate cancer before. Before the catheterisation, I had reduced sexual ability, no doubt. But now, there is no hope because I have a rubber [catheter] in there. My situation now has ‘moved from the frying pan into the fire’. (Ofori, patient)

Participants were open in talking about their sexual lives, especially during the follow-up interview. Some spouses expressed concerns about their husbands feeling less masculine because they were unable to have sex:

Hmmm, sex is not my main worry now but rather how he [husband] will be healthy so that we have a happy life together. But my husband is worried! He’s ashamed. Yes, I note he is unhappy about it because he asked how I feel about his situation [his inability to have sex]. (Okonore, Kwaku’s wife)

Anytime there is a small quarrel, then he says I’ve changed my attitude because he is now impotent. No! No! Far from that. He’s paranoid. I’m not the type of woman who will divorce their husband because of impotence. But there have been times I wondered whether he will ever be able to regain his erectile function. It’s a pity this has happened to us. (Stella, Ofori’s wife)

Stella and Okonore both indicated that lack of sexual activity was not a problem for them, but they were concerned about how this impacted on their husbands. Struggles with masculine identity could thus have wider impacts on the marital relationship.

### Masculinity and socio-cultural status/economic roles

The third factor that men living with advanced prostate cancer reported as having an impact on their masculinity was a change in their socio-cultural and economic roles. Traditionally, a masculine role in Africa includes being the breadwinner and provider for the family. Such beliefs are associated with status, dignity, being independent and being superior. The impact on men’s social status conflicted with the traditional socially constructed patriarchal roles as the dominant people in society:

. . . now the whole situation is like moving straight from the head of the family to the tail. Because when one is unable to do something for himself (care), he becomes like a child, needing to be cared for. (Boat, patient)

Boat speaks to feelings of having a diminished role in the family. The metaphor *from head to tail* indicates a dent in Boat’s sense of his status as a man and also a reversal of his socio-economic role as a *provider* for the family to one as someone for whom others provide.

The connections between physical appearance, body image, socio-cultural status and reputation were highlighted by participants. A man’s appearance defines his personal identity and degree of social status. One participant recounted:

I can’t put on my beautiful Kente ntoma (traditional Ghanaian wear) because the catheter and urine bag will be seen by others. Also, wearing the Kente will expose my extreme weight loss. I need to wear something that can conceal it (catheter) and cover my entire body and not expose it too much. So, wearing my favourite Kente is out of the option. (Samson, patient)

Samson has to change his lifestyle by abandoning the traditional costume he wears on special occasions because wearing it reveals his urinary catheter. Gyasi, like Boat, was unable to take care of himself without help, and implicitly is unable to take care of others. They both felt that their sense of masculinity is affected by moving to a subordinate role:

All that I can say is that I don’t feel like having that control over myself as a man as before. It is a bit of a challenge seeing myself moving from that level (high) at both work and home to a wheelchair life. I was in charge of giving instructions at our command [military], but now I have to follow instructions from others [at home]. (Gyasi, patient)

Men reported that their role as economic head of the family unit was lost:

. . . when my husband had prostate cancer, I knew the family was in trouble. That took the staple out of the family. My husband plays a central role in our family lives by providing for us. (Abiba, Maegyida’s wife)

it has affected every aspect of my life; that’s my work, marriage, and parental responsibilities. I can’t even pay my children’s school fees because of the hospital bills. This is untoward; because, as a man of the house, I’m supposed to do this and not the other way around. (Kwaku, patient)

Abiba and Kwaku describe difficult times for their respective families. The onset of advanced prostate cancer, therefore, disrupts the socio-cultural and economic status of men who are mostly heads of family units and providers to their immediate and sometimes extended family. For example, Abiba indicated that the expenses on her husband’s treatment nearly disintegrated the family (‘staple out of the family’).

### Masculinity and emotionality

Dealing with advanced prostate cancer – with the undesired physical changes it brings, the loss of libido and impotence and the loss of social and economic status – can have a strong emotional impact. Men may be socialised into the stoic acceptance of challenges; trying to live up to stereotypical presentations of masculinity, with associated consequences for men’s emotional lives. For example, men’s displays of emotions such as sadness, fear or vulnerability are all too often interpreted by society as signs of weakness or a failure to be a *real man*. Participants reported a reluctance to show signs of emotional weakness, which meant that for some men there was a reluctance to talk about or reveal emotional distress; this also affected their health-seeking behaviour:

[. . .] in the past, I had thought of ending my life when things were tough. I’ve suffered. I have. For a man like me to have no cedi (unit of Ghanaian currency) on me, it is a sad story [Tears dropping, interview paused as participant showed emotion in the interview]. (Kwaku, patient)

Kwaku’s experience illustrates the struggles some men had with loss of status and their emotional response to that. It also indicates how different aspects of masculinity, physicality, social status, sexuality and emotions are intertwined. To try and live up to the belief that *men don’t cry*, Kwaku had not revealed his emotional turmoil previously:

I couldn’t tell anyone about it. And no one asks me how I feel emotionally. But speaking to you the other time (during the first interview) and now, I felt I should share this with you and let off some ‘steam’. (Kwaku, patient)

He, however, shed tears during the interview, revealing his deep emotional turmoil and his need to let off steam. It is common in the Ghanaian language to hear the expression ‘barima ɛnsu’, which means *men don’t cry*. Men are expected to be brave and not cry or allow others to see their tears. Some of the participants, however, cried during the interviews and that may be a sign of previously suppressed emotions (as indicated by Kwaku above). Such norms of masculinity made some participants feel unable to express their emotions:

Occasionally I’m sad, but as a man, I have to hide some of those feelings from the children and their mother (Atta’s wife). But you can’t hide it all the time, they get to know and ask you about it. Sometimes I regret still living. (Atta, patient)

Atta’s case is typical in that he experienced difficult emotions at times yet tried to hide them in the belief that not doing so compromised his masculinity.

Relatives and caregivers noticed that participants were withdrawn or unhappy, but sometimes repeated the expectations that men should be strong and brave:

Of late he is not cheerful, not even a fake smile, and this makes us all sad. It’s difficult seeing him this way because he wasn’t like this . . . He has always been brave and endured strong emotions. (Sabi, caregiver of Boat)

I know I am sometimes emotional and do get angry at the least thing . . . and I know they [family] are not very happy about it. (Boat, patient)

Despite participants reporting efforts to hide feelings that are not associated with masculine ideals, men experienced such emotional turmoil that it could not always stay hidden. Sabi reported that Boat could no longer bear to hide his emotions, and this was noted by his family. The family was also saddened because Boat’s emotional outburst was unusual.

There was also some evidence that emotional stoicism and control had implications for health-seeking behaviour. Damaa showed a link between his notion of masculine norms and health-seeking behaviour as he did not seek help until his prostate cancer was at an advanced stage:

I’ve always been a strong man. I don’t fall sick often. So, I didn’t pay attention to my symptoms until I experienced severe pains and had to be taken to the hospital by my mother. Hmmm . . . the results broke my heart; the doctors said if I’d reported early, they could have done surgery to remove it, but it was too late now. (Damaa, patient)

Damaa’s belief in his strength as a man led to his late diagnosis of prostate cancer which had then spread to other parts of his body, making him weaker every day.

## Discussion

This article reports findings relating to ways in which men’s experiences of having advanced prostate cancer present challenges to men’s gender identity and to hegemonic notions of masculinity. We identify the importance of paying attention to multiple and sometimes contradictory masculinities that exist across cultural settings. This also offers a greater understanding of the nuances of advanced prostate cancer experiences among Africans living in the global south.

### Masculinity and changes in physical appearance

Our findings indicate that men become very conscious of their physical appearance. The challenges are significantly accentuated by the contexts that men are living in.^30,40^ For example, in resource-limited settings with difficulties in resourcing supplies such as incontinence pads and lack of access to medicines.^41^

Our participants reported that they previously had taken great pride in their physical appearance. Other research has highlighted the significant impact of feelings of shame or dishonour that men can face when hegemonic masculinity is under threat.^42,43^ Ouzgane and Morrell^44^ identify how appearance is central in demonstrating one’s social status or wealth, which is important in the African context. Given this context, *changing physicality of the body* deeply affected men’s sense of dignity, embodiment and image; the body is both material and presentational for men.^26,45^

Men in this study felt less masculine due to the physical changes following the diagnosis, and treatment side effects, of advanced prostate cancer. The use of physicality as a measure of one’s masculinity resonates with the findings of similar studies in Brazil,^46^ Australia^47^ and the United Kingdom.^48^ Most of these studies, however, emphasised *physical strength* alone and not *physical appearance* as was prominent in this study. For example, lack of physical strength made men feel vulnerable, and loss of strength contributes to a sense of incapability and vulnerability.^49^ Our findings on physical appearance are important because they add to knowledge and illustrate sociological nuances apparent in the ways in which masculine identities are affected by the experience of advanced prostate cancer in a different cultural and social context from existing studies.^21^ For instance, the experiences of incontinence leave men with a diminished sense of agency over their own bodies and feeds into notions of shame.

Family caregivers in this study expressed worry about men struggling with their gender identity and how this impact immensely on their own lives and the socio-cultural fabric of the family. Caregivers reported that men’s decline in physical appearance and strength contributed to their reduced erectile function, a diminished sense of masculinity and stoicism. This perspective contributes additional insight to the literature on masculinity and prostate cancer.

### Masculinity and sexuality

It is known that various degrees of erectile and sexual problems are common for men living with prostate cancer^50^ but particularly those living with advanced prostate cancer. There is very limited qualitative understanding of the implication of sexual problems on masculinity. Being masculine in this study was also conceptualised as ‘being whole’ physically without missing body part. Therefore, having one’s testes removed as a treatment option when managing advanced prostate cancer was unwelcome news. Most of the reasons for refusing such intervention were about how the removal of the testes could affect their sexual life and damage their masculinity. So, equating the absence of a testes to the belief that ‘no testes’ means ‘no longer a man’ have implications for deciding whether or not to accept treatment options such as surgical removal of the testes, which may be perceived as an affront to their masculine identity.^42^

Our findings demonstrate the importance of sexual potency for participants, a finding supported by other studies, for example.^20^ Adinkrah^42^ discusses how masculinity is cast in sexual terms. For men, sexual impotence can challenge their masculinity and a male sense of self-worth, such that Adinkrah observes impotence as a ‘major violation of masculine space and integrity’ (p. 475) that causes stigma.^51^

The use by some participants in this study of phrases such as my ‘manhood is not functioning’ and ‘losing my manhood’ shows a lack of power to fulfil a duty that is expected of men, a common masculine ideal in the Ghanaian context.^52^ Men in this study expressed concerns about their declining or lost sexual strength and how they socially construct this as being a ‘breach of masculinity’ and its effect on their patriarchal obligations. Our findings also resonate with Rivas *et al.’s*^22^ meta-ethnography of ethnic minority patients with prostate cancer, in Western contexts, that found erectile dysfunction was a significant concern and a particular challenge to men’s masculine identities. Unlike findings in Western contexts, however, participants in this study believed that their sexual potency should be everlasting. They therefore resort to buying medicines and herbal drugs, in attempts to regain virility. Aphrodisiacs often made their sexual potency, hence the use of the term by some participant ‘out of the frying pan into the fire’.

We interviewed some patients’ wives, and it was interesting that their concerns were not for themselves in terms of any impact on their sense of femininity or being desirable. Rather their concerns also focused on the impact that sexual difficulties have on their husbands. This represents a different perspective from that found elsewhere,^53,54^ as the wives were more concerned about men’s health. This study adds to limited literature addressing spousal perspectives about men’s experiences and the impact they observe this has on men’s gender identity. Spousal caregivers in this study tended to dismiss any sexual concerns not because it is not a major issue for them, but because they felt protective of their husband. The women seemed to prioritise bolstering their partners’ self-esteem and did not want to exacerbate challenges men are already dealing with, possibly aware of the cultural discourses about male sexual and masculine prowess. Women were also trying to live by cultural norms such as men’s assumption of a leadership role.

### Masculinity and socio-cultural status/economic roles

This study also highlights ways in which participants seek to maintain and live up to their socio-cultural status and economic roles in the family and their communities. Living with advanced prostate cancer brings about significant changes in men’s societal roles and social identities. For example, there are financial implications that come from no longer being able to work due to ill-health. In addition, health insurance excludes the cost of treating prostate cancer and other chronic diseases. Consequently, men, who are usually breadwinners of their families, are unable to discharge their financial obligations for their families amid the cost of treatment that can deplete their life savings. This also put some financial burden on the wider family who take on responsibilities to pay for the cost of treatment and other diagnostic investigations – a situation some men described as ‘moving down the social ladder’ with associated impacts on their self-esteem and masculine identity.

The challenges presented are significantly accentuated by the culture that men are living in. For example, men taking on the traditional role as the head of the household and being responsible for the financial needs of his family.^55^ This finding resonates with what Broom^13^ calls ‘cultural codes of masculinity’ (p. 73). Our findings concur with other studies in which men identify strongly with being their family’s ‘provider’ or ‘head of household’; not being able to fulfil this role or to live up to expectations can be very difficult for men to adjust to in reframing notions of hegemonic masculinity.^47,51,56^

Andoh-Arthur *et al.*’s^51^ work about a ‘burden of masculinity’ is relevant here. In a study attempting to understand the high rate of suicidal behaviour among Ghanaian men, they identify the damaging impacts that social expectations and definitions of masculinity can have on men and their families. Losing the ability to be the family breadwinner, potentially exacerbated by reliance on others for financial support, is akin to what Andoh-Arthur *et al.*^51^ identify as a ‘breach of patriarchal norms’ (p. 66).

### Masculinity and emotional response

The notion of emotional stoicism and control of men were evident in this study. Such notions make some men reticent about their health needs^46,57,58^ as it is assumed that men should be strong.^59^ Holding on to such a belief, some men were diagnosed late because they did not seek health support earlier as reported in previous studies.^60,61^ Once faced with serious illness, emotional stoicism also continues; it is considered unmasculine for a man to express or admit feelings of weakness and emotional dependency. Such hegemonic belief has implications for their help-seeking^62^ and their use of health services.^63^ Interestingly, in the study, the interviews seemed to provide an opening for men to reveal emotions they acknowledged hiding from those close to them – maintaining a masculine ideal of being stoic and not showing emotion. This has implications for health professionals probing and making room to discuss emotional issues that men with advanced prostate cancer may have. For example, one participant who had contemplated committing suicide opened up during the interview adding that ‘no one found out about his emotional state’.

This study found that men became emotional in the interview setting related to talking about (1) being unable to fulfil their socio-economic responsibilities, (2) losing patriarchal control and (3) being impotent or having a reduced sex drive. Yet some men made efforts to conceal their emotional problems from their families as a sign of bravery or not to make the family feel sad about their situation, as reported in other studies.^12,47,59,64^ For example, men in this study reported the belief that they were not supposed to cry (‘barima ɛnsu’) even if they are in pain, and this resonates with other studies that found the notion of hegemonic masculinity reinforced, with men holding on to the belief that it was not masculine to admit to weakness or to cry.^65^

When emotions are hard for men to express that may also exacerbate mental health problems and suicidal ideas,^56,66,67^ and thus further impact negatively on men’s health.^65^ Therefore, this study challenges the notion that men’s experience of loss of masculinity is principally physical and not social.^68^ This study provided a window for men to discuss the challenges that they have acknowledged hiding from others in order to maintain a masculine front. The additional perspectives of family caregivers, who play a central role in caring for men living with advanced prostate cancer, are critically important, however.

The additional perspectives of family caregivers provide deeper understanding of the experiences of the men within the family context. Caregivers expressed concern about the challenges of prostate cancer on men’s masculine identity, rather than for themselves or how the care is impacting their own lives. Specifically, spousal caregivers were not very concerned about, for example, the lack of sexual activity in their lives but demonstrated a solicitude of how this is impacting on their husbands’ sense of dignity and gender identity. Similarly, caregivers were keen to support men and ameliorate financial and other concerns for men as much as they could. The challenges men face to their masculine identity thus appear primarily embedded in attempting to live by forms of deeply embedded cultural norms of ‘hegemonic masculinity’.

### Reflections

Sharing sensitive information about masculinity and end of life experiences was sometimes an emotional experience for participants,^69^ as well as for the first author who conducted all interviews. Researchers dealing with such sensitive research need to ensure adequate support for participants and themselves, especially when confronted with a dilemma as a clinician-researcher.^70^ For example, observing participants using a care technique that is hazardous could be a challenging experience for a researcher who is clinically trained.

One particular strength of this study is its dyadic nature, presenting an opportunity to address the shared and individual concerns of the patients and their family caregivers.^66^ Hence, the planning of health interventions for men with advanced prostate cancer must factor caregivers and families, especially in such contexts in which health decision-making is a shared one within an acceptable social-cultural context.^61,67^

### Strengths and limitations

The findings report the researchers’ analysis of the participants’ experiences within a specific context, and we make no claims to the generalisability of the findings. Being a qualitative study, however, one of its greatest strengths is the range of issues uncovered, allowing the development of a deeper understanding of concepts and ingrained socio-cultural beliefs^71^. It also unravelled the issues that are believed to be sensitive to explore, highlighting the benefits of a second interview in terms of relationship-building between participant and researcher.

This study is novel in that other studies exploring the experiences of prostate cancer cover some but not all the concepts of physical appearance, sexuality, social values and emotionality. In this study, interviews with caregivers shed further light on the difficulties that men grappled with in confronting how advanced prostate cancer wrought changes to their masculine identities. The additional perspectives of family caregivers enhanced the understanding of the intersectionality of masculinity and experience of prostate cancer within a non-Westernised context.

## Conclusion

Understanding the impact of advanced prostate cancer on masculinity has been explored within Western contexts, and within this work, we present some knowledge about the particular impacts of prostate cancer on African men. There has been, however, less research exploring masculinity and prostate cancer in African contexts in which patriarchal-idealised gender roles are still strongly embedded.

This article has reported on the experiences of men and their caregivers in Ghana, with a focus on how men experience masculinity in a non-Western, patriarchal cultural and social context. The four themes identified (changes in physical appearance, sexuality, social status/economic roles and emotionality) all relate to challenges to men’s sense of masculinity. These are also interconnected and collectively affect men’s health and illness in a social setting underpinned by a strong social value of hegemonic masculinity.

Understanding the intersectionality and changes in masculinity could lead to increased occurrence of and a desire for an open and honest discussion on how masculinity is affecting men’s quality of care and to identify how these could be mitigated. Improving understandings of masculinity highlights the need to develop culturally appropriate as well adaptations made to important social and cultural values when delivering care.

## Supplemental Material

sj-docx-1-pcr-10.1177_26323524231176829 – Supplemental material for ‘Out of the frying pan into the fire’: a qualitative study of the impact on masculinity for men living with advanced prostate cancer Click here for additional data file. Supplemental material, sj-docx-1-pcr-10.1177_26323524231176829 for ‘Out of the frying pan into the fire’: a qualitative study of the impact on masculinity for men living with advanced prostate cancer by Yakubu Salifu, Kathryn Almack and Glenys Caswell in Palliative Care and Social Practice

## Declarations

**Ethics approval and consent to participate:** Ethical approvals were obtained from research ethics committees in the United Kingdom (reference no F12092016) and Ghana (reference no CHRPE/AP/496/16). Informed consent was obtained from all participants. There was no relationship between researchers and the participants, and only the first author had access to identifiable information.

**Consent for publication:** Participants provided written informed consent prior to the start of data collection.

**Availability of data and materials:** The data that support the findings of this study are available from the corresponding author upon reasonable request.
