# Supplementary material for: ‘Out of the frying pan into the fire’: a qualitative study of the impact on masculinity for men living with advanced prostate cancer
Source: Palliat Care Soc Pract. 2023 May 29;17:26323524231176829. doi: 10.1177/26323524231176829 (PMC10230602; doi:10.1177/26323524231176829)
Supplement: sj-docx-1-pcr-10.1177_26323524231176829 – Supplemental material for ‘Out of the frying pan into the fire’: a qualitative study of the impact on masculinity for men living with advanced prostate cancer [file sj-docx-1-pcr-10.1177_26323524231176829.docx]

**Consolidated criteria for reporting qualitative studies (COREQ): 32-item**

**Checklist**

| No. | Item Guide questions/description | **Reported on Page**  **#** |
| --- | --- | --- |
| **Domain 1: Research**  **team and reflexivity** |  |  |
| *Personal*  *Characteristics* |  |  |
| *1.*  *Interviewer/facilitator* | **Which author/s conducted the interview or focus**  **group?**  YS | Page 4-5 |
| *2. Credentials* | **What were the researcher’s credentials? E.g., PhD, MD**  YS –PhD, MPhil, BSc, RN, DipEd, DipN    KA – PhD, MA, BA  GC- PhD, MA, BA |  |
| *3. Occupation* | **What was their occupation at the time of the**  **study?**  YS registered nurse and  Lecturer  KA and GC- Sociologist, academics and Palliative care researchers in the UK | NA |
| *4. Gender* | **Was the researcher male or female?**  YS– male  KA and GC – females | NA |
| 5. Experience and  training | **What experience or training did the researcher**  **have?**  YS-has a certificate in qualitative research methods from the University of Alberta-Canada and published a number of qualitative research.  KA is a Professor, and GC is an independent researcher. They are both experienced qualitative researchers in palliative care with several publications |  |
| *Relationship with*  *participants* |  |  |
| 6. Relationship  established | **Was a relationship established prior to study**  **Commencement?**  YS- Is a clinician-researcher but did not have any direct prior contact with the participants.  KA and GC are also experienced, researchers who are non-clinician and brought in fresh, unbiased perspectives. |  |
| 7. Participant  knowledge of the  interviewer | **What did the participants know about the researcher? e.g. personal goals, reasons for doing the research**  Participants did not know researchers but were told of the goal of the research. |  |
| 8. Interviewer  characteristics | **What characteristics were reported about the**  **interviewer/facilitator? e.g. Bias, assumptions,**  **reasons and interests in the research topic**  These have been provided above. |  |
|  |  |  |
| **Domain 2: study**  **design** |  |  |
| *Theoretical framework* |  |  |
| 9. Methodological  orientation and Theory | **What methodological orientation was**  **stated to underpin the study? e.g.**  **grounded theory, discourse analysis,**  **ethnography, phenomenology, content**  **analysis**  This was a qualitative repeat study using a thematic analysis. | Page 4 |
| *Participant selection* |  |  |
| 10. Sampling | Sampling  How participants were selected? e.g.  Purposive sampling | Page 4 |
| 11. Method of approach | Face to face interviews (individual and dyads) | Page 4-5 |
| 12. Sample size | 46 (this includes all participants: men, and family caregivers) | Page 5 |
| 13. Non-participation | Four men did not take part in the second interview due to death | Page 4 |
| *Setting* |  |  |
| 14. The setting of data  collection | Where was the data collected? e.g.  home, clinic, workplace  For the men and their caregivers, the interviews occurred mostly at home and on few occasions in a private room at the hospital. | Page 5 |
| 15. Presence of nonparticipants | Was anyone else present besides the  participants and researchers?  Dyad interviews had the patient and caregiver, and the rest were individual interviews. |  |
| 16. Description of sample | What are the important characteristics  of the sample? e.g. demographic data, date  These have been provided on the stated pages. | Pages 4-6 |
| *Data collection* |  |  |
| 17. Interview guide | Were questions, prompts, guides provided by the authors? Was it pilot  tested?  Yes | See interview guide submitted as part of supplementary list |
| 18. Repeat interviews | Were repeat interviews carried out? If  yes, how many?  Yes; 2 for the men and their caregivers | Page 4-5 |
| 19. Audio/visual  recording | Yes | Page 5 |
| 20. Fieldnotes | Yes | Page 5 |
| 21. Duration | Average of 45 minutes for the interviews and 1. 30 minutes for the focus group | Page 5 |
| 22. Data saturation | Was data saturation discussed?  Yes | Page 4 |
| 23. Transcripts returned | Were transcripts returned to participants  for comment and/or correction?  No, this does not conform to the philosophical position of the researchers. |  |
| **Domain 3: analysis and**  **findings** |  |  |
| *Data analysis* |  |  |
| 24. Number of data  coders | How many data coders coded the data?  Three (YS, KA and GC). | Pages 6 |
| 25. Description of the  coding tree | Did authors provide a description of the  coding tree?  Yes a description of the coding provided | Page 5 |
| 26. Derivation of themes | Were themes identified in advance or derived from the data?  Themes were derived from the data | Page 6 |
| 27. Software | What software, if applicable, was used to manage the data?  EndNote, Nvivo | Page 5 |
| 28. Participant checking | Did participants provide feedback on the  findings?  No, this was not in line with author's philosophical underpinning |  |
| *Reporting* |  |  |
| 29. Quotations presented | Were participant quotations presented to  illustrate the themes/findings? Was each  quotation identified? e.g. participant  number  Yes. Pseudonyms were used to identify  each quotation and was fairly spread across | Pages 8 to 15 |
| 30. Data and findings  consistent | Yes | Pages 8-15 |
| 31. Clarity of major  themes | Yes-four major themes | Page 6 |
| 32. Clarity of minor  themes |  |  |
